# HIV type 1 drug resistance patterns among patients failing first and second line antiretroviral therapy in Nairobi, Kenya

**DOI:** 10.1186/1756-0500-7-890

**Published:** 2014-12-09

**Authors:** Peter Koigi, Musa Otieno Ngayo, Samoel Khamadi, Caroline Ngugi, Anthony Kebira Nyamache

**Affiliations:** Department of Medical Laboratory Sciences, Kenyatta University, Nairobi, Kenya; Center for Microbiology Research, Kenya Medical Research Institute, Nairobi, Kenya; Centre for Virus Research, Kenya Medical Research Institute, Nairobi, Kenya; Department of Medical Microbiology, Jomo Kenyatta University of Agriculture & Technology, Nairobi, Kenya; Department of Microbiology, Kenyatta University, Nairobi, Kenya

**Keywords:** Antiretroviral drug resistance, Resource-limited settings, Second-line therapy

## Abstract

**Background:**

The ever-expanding rollout of antiretroviral therapy in poor resource settings without routine virological monitoring has been accompanied with development of drug resistance that has resulted in limited treatment success.

**Methods:**

A cross-sectional study with one time viral load was conducted during the period between 2012 and 2013 to determine treatment failure and drug resistance mutations among adults receiving first-line (44) (3TC_d4T/AZT_NVP/EFV) and second-line (20) (3TC/AZT/LPV/r) in Nairobi, Kenya. HIV-1 *pol-*RT genotyping for drug resistance was performed using an in-house protocol.

**Results:**

A total of 64 patients were recruited (mean age 36.9 yrs.) during the period between 2012 and 2013 of the 44 adult patients failing first-line 24 (40.9%) had drug resistance mutations. Eight (8) patients had NRTI resistance mutations with NAMS M184V (54.2%) and K65R (8.4%) mutations being the highest followed by TAMs T215Y and K70R (12.5%). In addition, among patients failing second-line (20), six patients (30%) had NNRTI resistance; two patients on K103N and G190A mutations while V106A, Y184V, A98G, Y181C mutations per patient were also detected. However, for NRTI two patients had TAM T215Y. M184V mutation occurred in one patient.

**Conclusions:**

The study findings showed that HIV-1 drug resistance was significantly high in the study population. The detected accumulated resistance strains show that emergence of HIV drug resistance will continue to be a big challenge and should be given more attention as the scale up of treatment in the country continues.

## Background

The Kenyan ministry of health began providing antiretroviral (ARV) therapy to the public sector in 2003 [1]. Kenya through the President’s Emergency Plan for AIDS Relief program, has significantly promoted access to ART [1–3], with an estimated coverage of 52% (360,000 with CD4 count <350), of the total 694,024 people living with HIV by the end of 2010. The Kenya government aims to achieve universal access to ART by targeting 90% (760,316) patients of the total of 844,795 patients in need of ART, by the end of 2014 [4].

Until 2009, the standard first-line regimens were stavudine (d4T) plus lamivudine (3TC) combined with a third agent: a non-nucleoside reverse transcriptase (RT) inhibitor (NNRTI) in adults and older children or ritonavir-boosted lopinavir (LPV/r) in young children who had received nevirapine (NVP) for prevention of mother-to child transmission (PMTCT) [5, 6]. By 2010, the Kenya government adopted WHO recommendations for early initiation of ART based on medical criteria and patient’s capacity to treatment adherence (HIV drug resistance report 2010-2011). Stavudine (D4T) was replaced by tenofovir disoproxil fumarate (TDF) in adults and older children and abacavir (ABC) in younger children, respectively. Adults beginning ARV treatment increasingly received TDF rather than d4T for first-line therapy and children increasingly received ABC rather than d4T [7, 8].

With the introduction of combination therapy with potent and cost-effective generic antiretroviral therapies (ARTs), quality of life has significantly improved, transmission rates reduced and survival rates increased among HIV infected individuals [9, 10]. However, this benefit is often limited with development of HIV-1 drug resistance. Previous studies have shown diverse pathways across different HIV subtypes in development of drug resistance. This could affect cross-resistance and the potential use of specific second-line regimens [11]. This concern may be increased in developing countries where all HIV-1 subtypes exist while drug resistance reference data is based HIV-1 subtype B [12].

Although the information on drug resistance patterns of HIV-1 among treatment-exposed patients is crucial for the development of novel effective drugs, unfortunately there is no existing system that monitors patterns of drug resistance in patients failing therapy [13]. This study was therefore aimed at determining the drug resistance patterns that circulate among patients failing first and second line drugs and their impact on predicted treatment options.

## Methods

### Study design and sample collection

Following WHO recommendations [14] a cross-sectional study was performed and one-time HIV-1 RNA viral load and CD4 Counts determined among patients who met the following criteria: confirmed positive HIV status; ≥18 years old; being on a first-line ART for at least 12 months and consented to participate in the study. In addition, those patients failing second-line were also recruited. This study was approved by Kenya National Ethics Committee through the Kenya Medical Research Institute (KEMRI)/National Ethical Review Committee. During the period of March 2012 and March 2013, patients were consecutively enrolled from KEMRI/KNH HIV comprehensive clinics centre. This is one of the referral centres for drug resistance testing in the country that manages approximately ≥1000 patients.

A standardized questionnaire was used to collect demographic data that included age, sex, ARV use (name, and switch), education levels, marital status and drug adherence. Those patients who had treatment interruptions at the time of blood sampling were excluded from the study. Additionally, patients who transferred from other treatment clinics and those who defaulted but came back for treatment later were also excluded from the study. In addition, participants with a previous history of ART exposure for prevention of mother to child transmission (PMTCT) or for post-exposure prophylaxis (PEP) were excluded from the study. Three ml of blood samples were collected from each participant for routine CD4 count counts and the remnants centrifuged to obtain plasma, for viral load quantification and HIVDR testing.

### HIV-1 RNA quantification and CD4 counts

CD4 T cell counts were performed using a FACSCalibur flow cytometer (Becton-Dickinson, NJ) and expressed in absolute count [3]. Individual test results were reviewed to confirm the accuracy of the automated software analysis. Viral loads were determined using NucliSens EasyQ (Biome’rieux, Marcy l’Etoile, France), with a lower limit of quantitation of 50 (1.69 log10) copies/ml of plasma, according to the manufacturer’s instructions. This was done to confirm virological failure (above 1000 copies/ml) before drug resistance testing was performed [6].

### PCR and sequencing

Whole blood samples from patients failing first and second line ART were obtained and separated to obtain Peripheral blood mononuclear cells (PBMCs) by Ficoll-Hypaque density gradient centrifugation. Proviral DNA was extracted from the PBMCs using DNAzol (GIBCO BRL, Life Technologies) lysis and ethanol precipitation. Nested polymerase chain reaction (PCR) was performed using AmpliTaq Gold (Roche Molecular Systems, Branchburg, NJ). Briefly, HIV- 1 *pol* gene was amplified using the primers (RT18:5′GGAAACCAAAAATGATAGGGGGAATTGGAGG3′ and RT21 5′ CTGTATTTCTGCTATTAAGTCTTTTGATGGG 3′) and second round primers (RT 1: 5′ CCAAAAGTTAAACAATGGCCATTGACAGA 3′ and RT4: 5′ AGTTCATAACCCATCCAAAG 3′) in the second round [15]. Amplification was achieved using 1 cycle of 95°C for 10 min and 35 cycles of 95°C for 30 s, 60°C for 30 s, and 72°C for 1 min, with a final extension of 72°C for 10 min [3] PCR amplification was confirmed by visualization with ethidium bromide staining of the gel. Positive generated amplicons were then directly sequenced using Big Dye technology on ABI 310 (Applied Biosystems, Foster City, CA) [16].

### Genotypic drug resistance analysis and HIV subtyping

Genotypic drug resistance in the pol-RT region was defined as the presence of one or more resistance-related mutations, as specified by the consensus mutation figures of the International AIDS Society-USA [3]. HIV subtyping tools, genotype (http://www.ncbi.nlm.nih.gov/projects/genotyping/formpagex.cgi), RIP (http://www.hiv.lanl.gov/content/sequence/RIP/RIP.html), and Jumping HMMER (http://jphmm.gobics.de/submission_hiv.html), were used in confirming subtyping [16].

## Results

### Demographic characteristics

A total of sixty four (64) patients failing first-line 44 (67.2%) and second line 20 (32.8%) therapy were evaluated. The participants were aged between 11 yrs and 70 yrs, with an average age of 37 yrs. Thirty (46.2%) were female and 34 (63.8%) males with all being residents of different regions of Nairobi. Data on length of time on the failing regimen was not available.

### Virological and immunological failure

Immunological and virological failure was determined by use of CD4 count (>350 cells/ml) and viral load (>1000 HIV RNA copies/ml). We determined whether age, marital status, WHO disease stage and education levels influenced virological and/or immunological failure (Table 1).

**Table 1:** **Baseline characteristics of HIV-1 infected Kenyan patients at enrolment**

		Gender		
Characteristics	All (n = 60)	Female (n = 30)	Male (n = 30)	P = value
Age (Years)	36.9			
**CD4+ count (cells/mm3)**				
**Mean**	230	207	243	
Range	(1-628)	(5-584)	(1-628)	
<100		6	9	P > 001
101-200		13	8	
201-300		1	3	
301-400				
401-500		4	5	
>600		2	1	
**WHO Disease staging**				P > 001
WHO Stage 1	9	7	2	
WHO Stage 2	23	11	12	
WHO Stage 3	24	12	12	
WHO Stage 4	4	-	4	
**Unstructured treatment interruptions**	39			
**Switch ART**	25			
Mean duration since infected (Months)	6.91 yrs			
First-line	42	21	21	
Second-line	18	9	9	
**Education levels**				
Primary	14	8	6	*P >* 0.001
secondary	22	9	13	
College/Tertiary	21	8	13	
None	2	1	1	
**Marital status**				
Single	5	3	2	*P >* 0.841
Married	49	20	29	
Separated/Divorced	5	4	1	
**Age groups**	21-30	4	5	P >0.001
	31-40	13	12	
	41-50	10	11	
	51-60	2	1	
	61-70		1	
**Sub-Total**		**30**	**34**	

### Drug resistance

WHO recommends two NRTIs and one NNRTI for first line drugs with a switch to protease inhibitor for a second-line therapy (Table 2). In this study, most patients on first-line ART were on Nevirapine (40.6%) containing regimens followed by Efavirenz 18(27.7%) combination with those on second-line being on Lopinavir (30.1%) containing regimen (Table 2). However, drug combinations among patient on first-line were; 18(27.7%) 3TC/AZT/NVP and least 3(6.8%) 3TC/TDF/NVP while 9(45%) 3TC/AZT/LPV/r and 1(5%) DDI/ABC/LPV/r were on second-line (Table 2).

**Table 2:** **Non nucleoside RT inhibitor (NNRTI) resistance mutations: percentages occurrence in patients treated with duo nucleoside RT inhibitor plus NNRTI first -line antiretroviral (ARV) regimens**

ARV Regimen		n.	103	106	181	188	190	A98	V108	Y184	K70
NNRTI	NRTI		NS	M	C	LCH	ASEQ	G	IV	V	KT
		60	n (%)	n (%)	n (%)	n (%)	n (%)	n (%)			
**Efavenz (EFV) containing regiments**											
**EFV**	TDF/3TC	3	1	-	1 (33.3)	-	1 (33.3)			1 (33.3)	1 (33.3)
	AZT/3TC	15	5 (33.3)	-	-	-	-				
**Sub-Total**		**18**	6 (30)	-	1 (5.6)	-	1 (5.6)				
**Nevirapine (NVP) containing regiments**											
**NVP**	D4T/3TC	4	-	-	-	-	-	-			
	TDF/3TC	3	2 (66.7)	-	-	-	1 (33.3)	-			
	AZT/3TC	18	1 (11.1)	-	-	1 (5.6)	2 (11.1)	-			
**Sub-Totals**		**25**	3 (19.2)	-	-	1 (3.8)	3 (11.5)	-			
**PI**											
**Lopinavir (LPV)/Ritinovir containing regiments**											
**LPV/R**	ABC/DDI	1	-	-	1 (100)	-	-	1 (100)			
	ABC/3TC	2	-	-	-	-	-	-			
	AZT/3TC	7	-	-	-	-	-	-			
	AZT/DDI	5	1 (20)	1 (20)	-	-	-	-	1 (20)		
	TDF/ABC	2	1 (50)		-	-	1 (50)	-		1	
**Sub-Totals (n = %)**		**17**	**2 (10)**	**1 (5)**	**1 (5)**	**-**	**2 (10)**	**1 (5)**		**2**	
**Totals**		**60**	**11 (18.3)**	**1 (1.6)**	**2 (3.2)**	**1 (1.6)**	**6 (9.4)**	**1 (1.6)**	**1 (1.6)**	**1 (1.6)**	**1 (1.6)**

Of the 44 patients on first-line, 18 (40.9%) had developed drug resistance mutations. The most common NRTI mutation was M184V 12 (66.7%) of the 18 patients who were infected with drug resistance strains. The K65R mutation occurred in two patients without TAMs in combination with M184V mutations among those on TDF containing first-line drugs. Of the 6 patients who harboured TAMs mutations, only 3 of them had T215Y mutations while 3 had K70R mutations among those who were on or on a previous AZT treatment. Nevertheless, these two mutational pathways did not occur both in a single patient (Table 3).

**Table 3:** **Nucleoside RT inhibitor (NRTI) resistance mutations: percentages occurrence in patients treated with duo nucleoside RT inhibitor plus NRTI first -line**

ARV regimens		n/60	NAMs nucleoside analogue mutations		Thymidine analogue mutations	
			K65R	184 V	T215Y	K70R
NNRTI	NRTI		R	Y/M	T/Y	R
			n (%)	n (%)	n (%)	n (%)
**Efavenz (EFV) containing regiments**						
**EFV**	TDF/3TC	3	1 (11.1)	1 (33.3)	-	1 (33.3)
	AZT/3TC	15	-	5 (33.3)	1 (6.7)	1 (6.7)
**Total**		**18**	**1 (5.6)**	**6 (33.3)**	**1 (5.6)**	**2 (11.1)**
**Nevirapine (NVP) containing regiments**						
**NVP**	D4T/3TC	4	-	2 (50)	-	1 (9.1)
	TDF/3TC	3	1 (10)	1 (10)	-	
	AZT/3TC	18	-	3 (12.5)	2 (8.3)	-
**Totals**		**25**	**1 (3.8)**	**6 (24)**	**2 (7.7)**	**1 (3.8)**
**PI**						
**Lopinavir (LPV)/Ritinovir containing regiments**						
**LPV/R**	ABC/DDI	1	-	-	-	-
	ABC/3TC	2	-	-	-	-
	AZT/3TC	7	-	-	-	
	AZT/DDI	5	-	-	-	-
	TDF/ABC	2	-	-	2 (100)	-
	AZT/TDF/3TC	1	-	-	-	-
**Sub Total**		**18**	**-**	**1 (5)**	**2 (10)**	-
**Totals**		**60**	**2 (4.7)**	**13 (21.7)**	**5 (7.8)**	**3 (4.7)**

The NNRTI mutation profiles differed between patients failing EFV compared to those on NVP containing regimens. The K103N 11(61.1%) was most common NNRTI mutations with highest frequency occurring on EFV (n = 6; 60%) than NVP (n = 4; 40%). Nevertheless, this occurrence was not any significant different (*p = 0.31*).

EFV seemed to select for a wider range of mutations compared to NVP (Table 2). Of the selected NNRTI mutations, majority of them occurred in 3TC containing regimen (Table 2).

Of the 20 patients failing second line drugs 6 patients harboured drug resistance mutations. These mutations were M184V (n = 1), T215Y (n = 2), V106A (n = 1), A98G (n = 1), Y18C1C (n = 1), G190A (n = 1) and V108IV (n = 1). However four patients had diverse drug mutations combinations of K103N and V108IV (n = 1); Y184V, G190A and T215Y (n = 1); K103N, M184V and T215Y (n = 1); and A98G, Y181C (n = 1) mutations. Single occurring mutations of V106A occurred as expected in a patient who had NVP regimen.

### HIV subtype

From the 64 samples that were successfully amplified and sequenced, analysis of the sequences showed that the majority belonged to HIV subtype A 35/64 (54.5%) followed by subtype D 17/64 (26.7%), subtype C 8/64 (12.5%) and CRF AE 3/64 (4.7%).

## Discussion

In this study, among patients who had virological failure with known NNRTI and NRTI mutations, those on first and second-line therapy were 40.9% and 30% respectively. The mutation patterns observed in this study were similar to those obtained in subtype B. The M184V was the most predominant mutation followed K103N. These findings were similar to those obtained from previous studies in Kenya [17] suggesting a consistent increase in exposure to this drug. Though most patients were on 3TC as first-line drug, majority of these patients failed due to the selected of M184V mutation. In addition, K103N mutation selected for NNRTI was also detected with high frequency in combination with M184V. This mutation is known to limit Nevirapine and Efavirenz drug efficacy. These findings were similar to those obtained from most poor resource settings [17–19]. Since over 73.4% patients were exposed to thymidine NRTIs and Lamivudine, This explains the number of NRTI related mutations observed in this study. Nevertheless, the most frequent mutations in RT were associated to lamivudine and NNRTIs exposure, which confirms the low genetic barrier of these drugs [20, 21].

Of the patients on first-line, 45.5% of them had no mutations associated with drug resistance similar to those reported in 17% South Africa [7], 47.3% Kenya [17]. The high rates could suggest possible better drug adherence or occurrence of non-drug associated mutations and probably outside the target sites. Determination of the presence of NAMs or TAMs drug resistance mutations among patients on first-line is usually conducted in predictive for second-line drugs. For instance, in this study, a K65R mutation was detected in two patients who were on TDF/ EFV treatment. This mutation tends to limit treatment with DDI, tenofovir and abacavir if previously not exposed [22]. This mutation also occurred in combination with M184V thus reducing susceptibility to TDF and EFV [22]. Contrary to previous studies recorded high rates of TAMs, this study had low TAMs rates also occurring singly signifying tolerability of these drugs and better adherence.

The occurrence of NNRTI mutations varied depending on the NVP and EFV regimen. Though patient were on NVP or EFV they both selected, K103N, Y181C, A190S mutations with mutations; Y188L, A190A detected on those on NVP while Y184V, K70KT mutations on those on EFV. Though mutations Y181C and G190A are mostly selected by NVP they were detected among those on EFV. These mutations Y181C and G190A are known to reduce susceptibility to Etravirine which could be used either for second or third-line regimen [11].

However, among patients failing second-line, 70% patient harboured virus with no drug associated mutations. This also suggested better patient’s adherence. In 20% (6/20) patient harbouring drug resistance viral strains, two patients had first TAMs mutation pathway of T215Y and also with NAMs M184V mutations. For NNRTI mutations, two patients had K103N and A190A. The observed low rates of acquired drug resistance among patients failing second-line suggested the currently used NRTI regimens are still effective. It could be argued that the detected mutations among patients on second line could be associated with the effect of persistent of acquired HIV-1 drug resistance mutations before switching to therapy.

Despite these findings, this study had limitations. The cross-sectional study and single sampling approach could not confirm virological failure or rule out blips, which can occur even during effective treatment [17–24]. Based on this study design, this study this finding could not be generalised to present the overall efficiency of the national ART program. Nevertheless, the amplified partial reverse transcriptase gene could present the entire *pol* gene that that is targeted by currently used drugs. Early Warning Indicators are often required as indicated by for efficient follow out of the ART and limitations of the development of HIV drug resistance. However, this study did not capture this data, including on length of time the patients were put on the failing regimen.

## Conclusion

This study detected minority complex drug resistance profiles that are predictive of resistance to currently used second-line NRTIs and NNRTIs regimens. Though the currently used drugs are still effective, the accumulated resistance strains observed in this study clearly shows that emergence of HIV drug resistance will continue to remain a priority while scaling up ART coverage.
